# Brainstem Cavernous Malformations Management: Microsurgery vs. Radiosurgery, a Meta-Analysis

**DOI:** 10.3389/fsurg.2021.630134

**Published:** 2022-01-10

**Authors:** George Fotakopoulos, Hugo Andrade-Barazarte, Juri Kivelev, Mardjono Tjahjadi, Felix Goehre, Juha Hernesniemi

**Affiliations:** ^1^Department of Neurosurgery, Henan Provincial People's Hospital, Zhengzhou, China; ^2^Department of Neurosurgery, University Hospital of Larissa, Larissa, Greece; ^3^Department of Neurosurgery, Helsinki University Central Hospital, Helsinki, Finland; ^4^Department of Surgery, Atmajaya Catholic University of Indonesia, Jakarta, Indonesia

**Keywords:** brainstem cavernous malformations, microsurgery, stereotactic radiosurgery, outcomes, management

## Abstract

Given the rareness of available data, we performed a systematic review and meta-analysis on therapeutic strategy microsurgical resection and stereotactic radiosurgery (SRS) for brainstem cavernous malformations (BSCMs) and assessed mortality, permanent neurological deficits (PNDs), rebleeding rate, and patients who require reintervention to elucidate the benefits of each treatment modality. Preferred reporting items for systematic reviews and meta-analyses (PRISMA) were used for protocol development and manuscript preparation. After applying all inclusion and exclusion criteria, six remaining articles were included in the final manuscript pool. In total, this meta-analysis included 396 patients, among them 168 patients underwent microsurgical treatment and 228 underwent SRS. Findings of the present meta-analysis suggest that regarding the total group of patients, in terms of mortality, late rebleeding rate, and PNDs, there was no superiority of the one method over the other. Applying the leave-one-out method to our study suggests that with low robust of the results for the bleeding rate and patients who require reintervention outcome factor, there was no statistical difference among the surgical and SRS treatment. Microsurgical treatment of BSCMs immediately eliminates the risk of rehemorrhage; however, it requires complete excision of the lesion and it is associated with a similar rate of PNDs compared with SRS management. Apparently, SRS of BSCMs causes a marked reduction in the risk of rebleeding 2 years after treatment, but when compared with the surgical treatment, there was not any remarkable difference.

## Introduction

Cerebral cavernous malformations (CMs) or cavernomas have a documented prevalence of 0.1–4% among the general population and represent 8–15% of all cerebrovascular lesions (1–3). The majority of CMs are found supratentorial, but ~20% of them are located in the brainstem (2, 4–6).

In general, the risk of intracerebral hemorrhage (ICH) of CMs depends on its clinical presentation [ICH or focal neurological deficit (FND) vs. incidental finding] and its location (brainstems vs. other locations) (7). Thus, brainstem cavernous malformations (BSCMs) have a 5-year estimated risk of ICH of 8% and 30.8% when presenting without or with ICH or FND, respectively (7). Moreover, repeated hemorrhages may cause severe and irreversible neurological deficits as a consequence of the critical functions of the brainstem. Therefore, these entities should be considered as critical diseases which require further management.

The optimal treatment strategy for BSCMs remains controversial. Microsurgical resection described as the first treatment modality for BSCMs has experienced marked improvement during the last decades, due to advances in neuroanesthesia, intraoperative monitoring, neuronavigation, imaging techniques, and postoperative care, with several surgical series reporting good outcomes (8–13). However, it carries high morbidity (up to 35%), requires complete resection of the lesion, and is limited to lesions extending to the ependymal or pial surface (8–13).

On the other hand, stereotactic radiosurgery (SRS) has arisen as alternative treatment modality to BSCMs (14–16). Since it apparently induces vasculature obliteration that decreases the subsequent risk of ICH, it can be applied to all patients regardless of general condition and comorbidities (17, 18). However, its efficacy over the long term is debatable.

Given the paucity of available data, we performed a systematic review and meta-analysis on both therapeutic strategies and assessed mortality, permanent neurological deficits (PNDs), bleeding rate, and patients who require reintervention to elucidate the benefits of each treatment modality.

## Materials and Methods

### Literature Search Strategy

A research protocol was developed in advance and detailed all aspects of the conduct of this meta-analysis. Preferred reporting items for systematic reviews and meta-analyses (PRISMA) were used for protocol development and manuscript preparation (19). The search of the literature was conducted using the PubMed, Cochrane, Ovid, and MEDLINE databases (last search on May 10, 2020). For the search, we used the appropriate keywords and MeSH terms as follows: brainstem, brainstem malformations, brainstem hemorrhage, brainstem management, BSCMs, and brainstem cavernoma.

Additionally, we limited our search to classical articles, clinical studies, clinical trials, controlled clinical trials, and multicenter studies. Also, the reference lists of the retrieved publications were manually examined to identify other potential qualified manuscripts that should be included. This process was repeated until no more articles were identified.

### Inclusion and Exclusion Criteria

If an article met the following criteria, PICO—population, intervention, comparison, other, it was considered eligible for incorporation into the current meta-analysis: (1) population: limited to patients with BSCMs presenting with ICH or FND; (2) intervention: only surgical or microsurgical and radiosurgery techniques were used for the treatment of BSCMSs; (3) comparison: to compare the outcomes between two techniques, to demonstrate at least one of the studied outcomes; (4) other: the full-text article was published in English; all articles refer to human adults.

**Figure 1:**
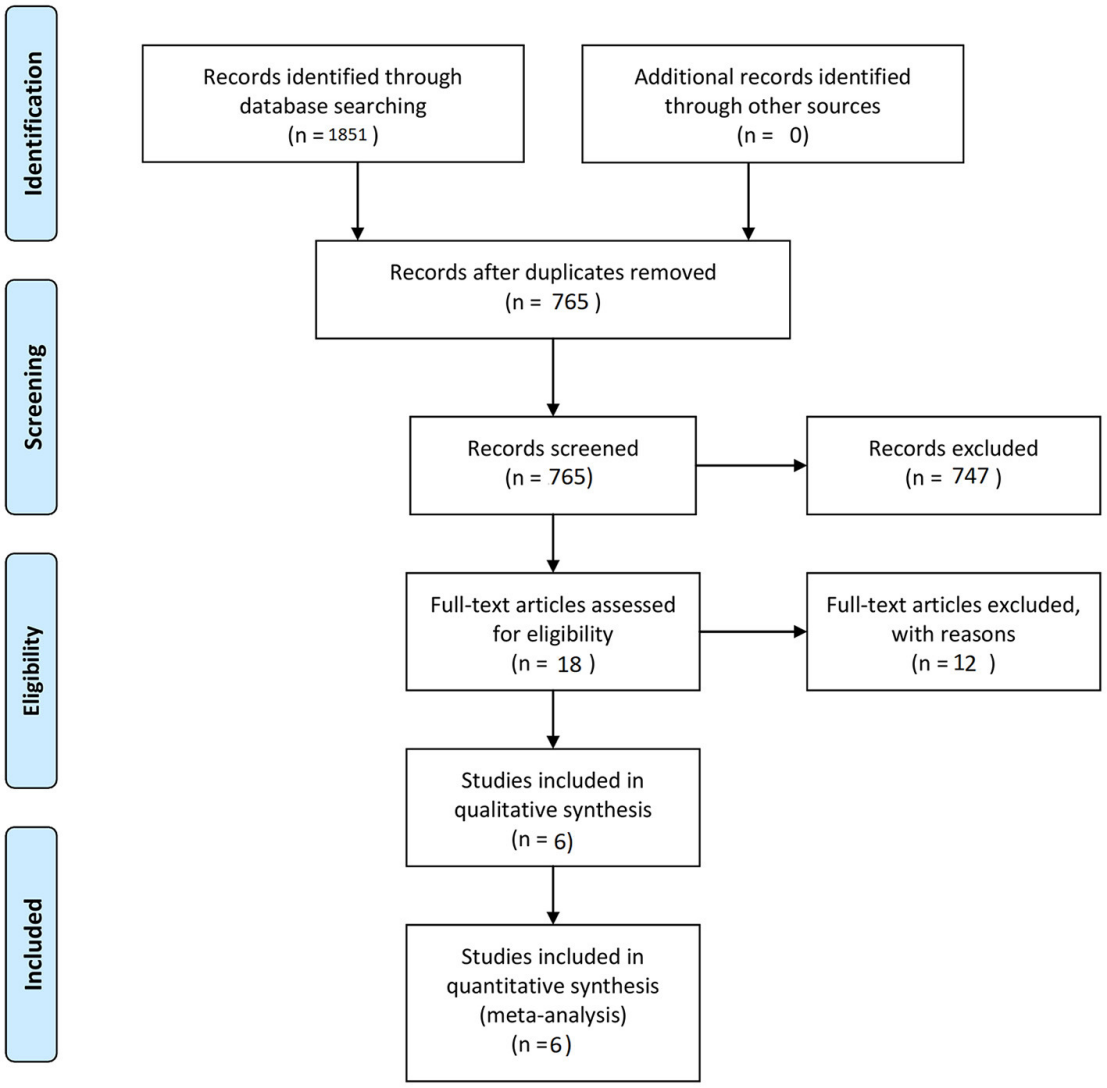
PRISMA flow diagram of search strategy and study selection.

The final aim was to collect a homogenous pool of manuscripts, which would highlight the results of surgery and SRS of BSCMs. Manuscripts were excluded from the article pool when focused on reoperation management, case reports, systematic reviews, unrelated outcome, comorbidities, experimental techniques, or one of the two techniques and all those which demonstrated mixed or unclear results. Twelve references with unclear or mixed results or were not written in English language were excluded from the 18 references in eligibility. The final article pool contained 6 manuscripts (9, 11, 20–23) (Table 1), which fulfilled our inclusion criteria and were considered appropriate for this meta-analysis (Figure 1).

**Table 1:** Design and baseline characteristics of included trials.

**Trial, year**	**Sample size**		**Mean age (year)**		**(%) males**		**PND**		**ReBl**		**LaReBl**		**ReInt**		**Mortal**	
	**Su**	**SRS**	**Su**	**SRS**	**Su**	**SRS**	**Su**	**SRS**	**Su**	**SRS**	**Su**	**SRS**	**Su**	**SRS**	**Su**	**SRS**
Porter et al. (11)	86	3	37	37	38	38	10	0	3	3	0	0	3	3	7	0
Mathiesen et al. (9)	22	5	–	–	42	–	5	1	3	2	0	2	0	0	0	0
Lunsford et al. (23)	17	49	39	39	56	56	0	14	17	22	0	5	0	4	0	2
Kida et al. (22)	8	140	35.6	38.6	–	–	–	4	0	10	–	–	–	–	0	0
Frischer et al. (20)	24	23	37.4	43.7	–	–	1	2	8	2	3	1	3	0	0	0
Haciyakupoglu et al. (21)	11	8	33.6	33.3	54	62	0	1	0	2	0	0	0	1	0	0

*Mortal, postoperative mortality; PND, permanent neurologic deficit; ReBl, rebleeding; LaReBl, late rebleeding; ReInt, re-intervention; Su, surgical; SRS, radiosurgery*.

### Data Extraction and Outcomes

Two of the authors independently extracted data from the included studies. The following essential information was collected: first author name, publication year, sample size, study design, outcomes, and other relevant data such as patient characteristics and literature quality scores. The articles were ordered and evaluated given the eligibility criteria (discrepancy was resolved through discussion with a third coauthor).

To compare both treatment modalities, we collected outcome measurements such as early postoperative mortality (within the first 30 days after treatment), PND (new neurological deficits that had not resolved by follow-up review at least 2 months after the intervention), early (within the first 30 days after treatment) and late rebleeding rates (2 years after treatment), and the need for reintervention (for BSCMs treated either by microsurgery or through SRS; Table 2).

**Table 2:** Meta-analysis results.

**Outcome**	**Trial**	**Groups**		**Overall effect**			**Heterogeneity**	
		**Su**	**SRS**	**Effect estimate**	**CI 95%**	***P*-value**	***I*^2^ (%)**	***P*-value**
PND	5	16	22	0.89	−1.92 to 0.14	0.09	−68.54	0.71
ReBl	6	31	41	0.24	−0.80 to 1.28	0.65	38.64	0.18
LaReBl	5	3	8	0.57	−1.67 to 0.53	0.31	43.31	0.12
ReInt	4	6	8	−1.29	−3.22 to 0.64	0.19	48.02	0.10
Mortal	6	7	2	−0.21	−1.67 to 1.25	0.78	−89.42	0.76

*I^2^ shows the percentage of total variation across studies that is due to heterogeneity rather than chance; CI, confidence interval; mortal, postoperative mortality; PND, permanent neurologic deficit; LaReBl, late rebleeding; ReInt, reintervention; ReBl, rebleeding; Su, surgical; SRS, radiosurgery*.

Postoperative outcomes reported by the included articles were assessed at least 6 months after the hemorrhage or intervention (surgery or radiosurgery). Additionally, to decrease the risk of bias in our manuscripts' pool, we used the quality assessment tool Newcastle Ottawa Scale (NOS) (24) (Table 3).

**Table 3:** Newcastle Ottawa Scale (NOS) quality assessment of final article pool.

**Trial, year**	**Study design**	**Newcastle ottawa scale**			
		**Selection**	**Comparability**	**Exposure**	**Total scores**
Porter et al. (11)	Retr	1	2	2	5
Mathiesen et al. (9)	Retr	2	3	2	7
Lunsford et al. (23)	Retr	1	2	2	5
Kida et al. (22)	Retr	2	2	3	7
Frischer et al. (20)	Retr	3	3	3	9
Haciyakupoglu et al. (21)	Retr	3	2	3	8

*Retr, retrospective; Prosp, prospective*.

### Statistics

Data from all included studies were used to evaluate the clinical features and neurological outcomes for patients who underwent microsurgical or radiosurgical management for BSCMs. We calculated log of odds ratios (log ORs) with 95% confidence intervals (CIs) and *p*-values for all evaluated independent variables.

Dichotomous outcomes were based on a meta-analysis, using the calculation of the log OR, with 95% CI. Log OR is defined as decimal logarithm of the odds of an event occurring in the surgical group, divided by the odds of the same event occurring in the SRS group. Log OR values <1 support microsurgery. Statistical significance is identified when *p* < 0.05, which provided that value 1.0 is not included in 95% CI. We investigated heterogeneity among studies through the *Q* test and quantified by the *I*^2^ statistic, which represents the percentage of total variation across studies with a predefined *I*^2^ > 50% as the cutoff point of statistical heterogeneity (24, 25).

### Assessment of Heterogeneity

We assessed heterogeneity by inspecting the graphs and the use of chi-square, *p*-value, and *I*^2^ statistics. We considered that a *p*-value of less than 0.1 is significant. We interpreted the *I*^2^ value of 50% or greater as high heterogeneity. The sources of heterogeneity were recognized using L'Abbé plots. In the case of heterogeneity, the random-effects model was used if there was high heterogeneity between studies and the leave-one-out model. Otherwise, the fixed-effects model was used. The results were visualized using OR forest plots. Furthermore, subgroup analysis was carried out to evaluate the impact of the preoperative condition on the results. The interaction tests were applied to test for differential effects of radiosurgery across subgroups. Publication bias was estimated according to the Egger test and visualized using funnel plots. The sensitivity analysis was performed in accordance with the leave-one-out method.

## Results

### Search Strategy

Initially, we identified a total of 18 potentially eligible articles. After applying all inclusion and exclusion criteria, six remaining articles were included in the final manuscript pool (9, 11, 20–23) (Figure 1). In total, this meta-analysis included 396 patients, among them 168 patients underwent microsurgical treatment and 228 underwent SRS (Table 1).

### Permanent Neurological Deficits

**Figure 2:**
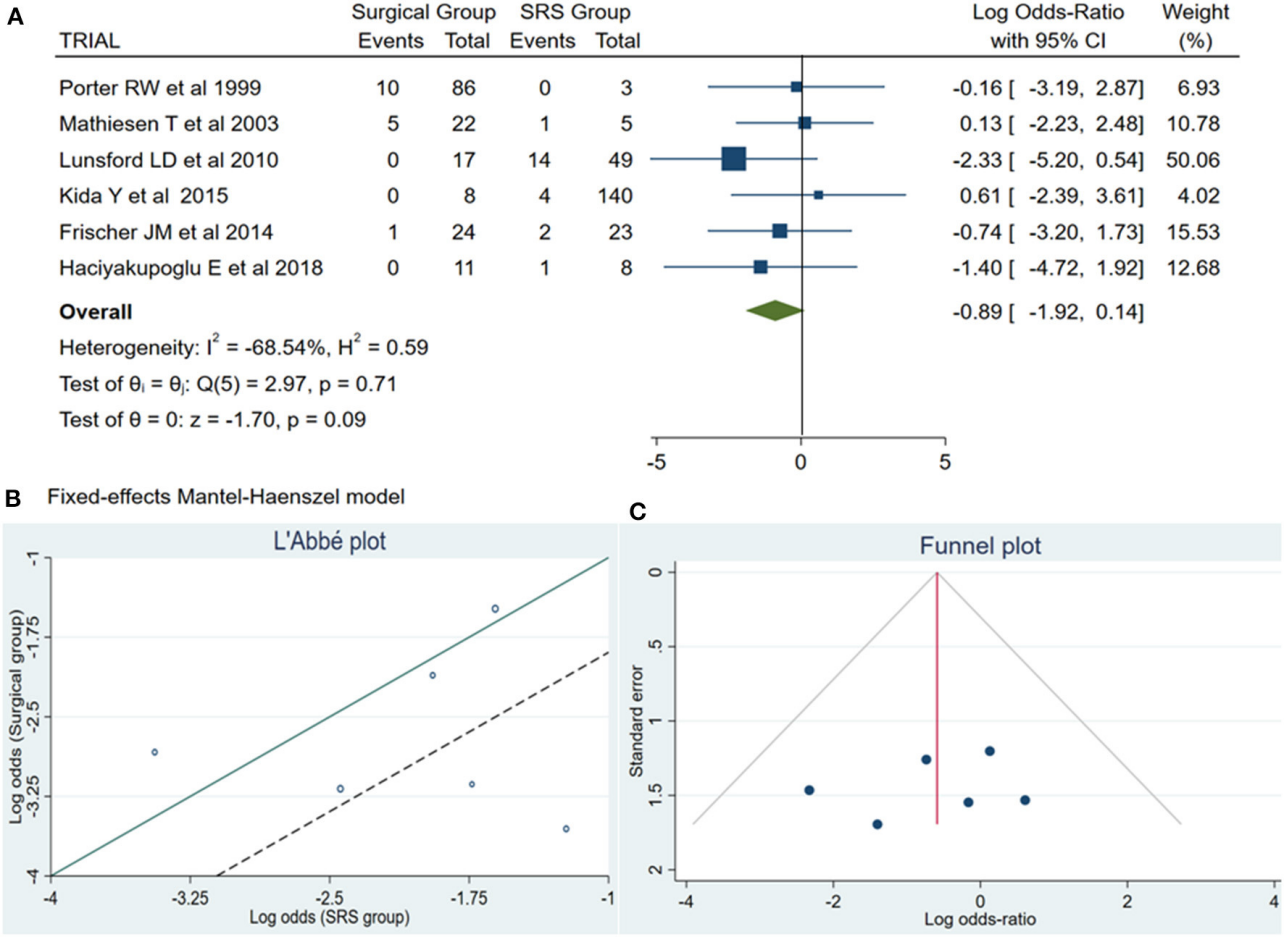
**(A)** Forest plot permanent neurologic deficit (PND): Results demonstrate no statistically significant difference between the two groups [decimal logarithm of odd ratio – log OR −0.89, CI 95%, 0.14 to −1.92, and *p* = 0.09]. **(B)** L'Abbé plot and contribution to overall heterogeneity (*p* = 0.71 and *I*^2^ = −68.54%). **(C)** Funnel plot, testing the sensitivity with funnel plot for PND, no statistically significant superiority existed between both groups, with low publication bias. SRS, Radiosurgical group; PND, permanent neurologic deficit; *I*^2^ shows the percentage of total variation across studies that is due to heterogeneity rather than chance; CI, confidence interval.

Of the total meta-analysis patient's cohort, 22 patients undergoing SRS reported PND, whereas 16 patients of the microsurgical group suffered from PND. The pooled results demonstrated a statistically significant difference between both treatment groups (log OR −0.89, CI 95% −1.92–0.14, and *p* = 0.09) with no heterogeneity (*p* = 0.71 and *I*^2^ = −68.54%; Figure 2).

### Rebleeding

**Figure 3:**
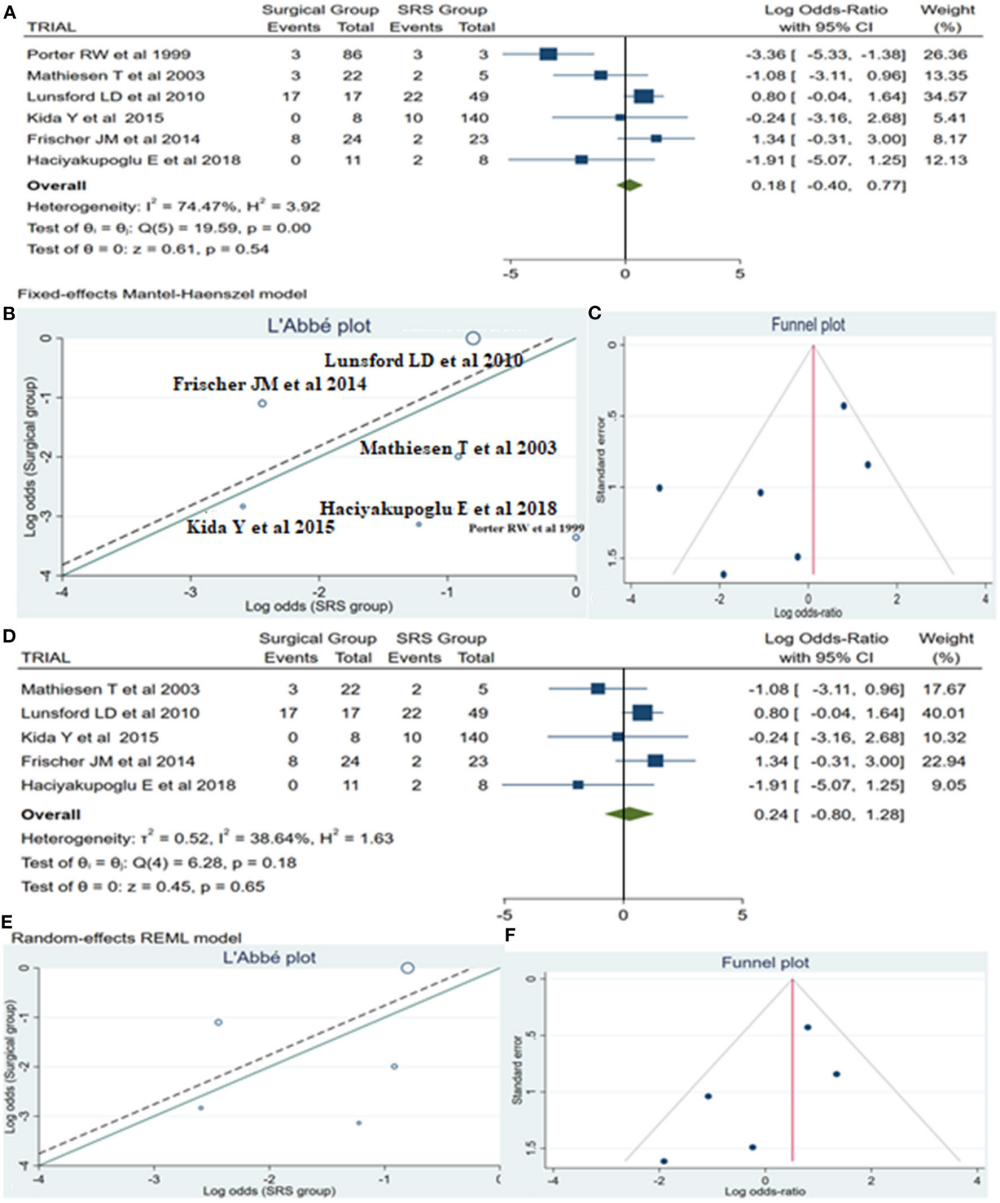
Rebleeding (ReBl): **(A)** OR forest plot ReBl: Results demonstrate no statistically significant difference between the two groups [decimal logarithm of odd ratio—log OR 0.18, CI 95% −0.40 to 0.77, and *p* = 0.54]; **(B)** L'Abbé plot and analysis of heterogeneity (*p* < 0.1 and *I*^2^ = 74.47%); **(C)** Funnel plots for publication bias on the available evidence relevant to BSCMs management; **(D)** OR forest plot ReBl without “Porter RW” (11) article: Results demonstrate no statistically significant difference between the two groups (log OR 0.24, CI 95% −0.80 to 1.28, and *p* = 0.65); **(E,F)** L'Abbé and funnel plots demonstrated very low heterogeneity (*p* = 0.18 and *I*^2^ = 38.64%) and no publication bias of the ReBl in groups of patients with BSCMs, respectively. SRS, Radiosurgical group; *I*^2^ shows the percentage of total variation across studies that is due to heterogeneity rather than chance; CI, confidence interval.

Information regarding rebleeding was available from all articles, comprising a total of 72 patients (31 from the surgical group and 41 from the SRS group). Initially, between these two methods existed no statistically significant (log OR −0.18, CI 95% −0.40 to 0.77, and *p* = 0.54), with high heterogeneity (*p* = 0.00 and *I*^2^ = 74.47%) (Figure 3A). However, looking at the L'Abbé plot (Figures 3B,E) and applied the leave-one-out method, we examined all possible combinations by removing one or two studies at the time and choose the combination without heterogeneity with the higher sample size and the less article removed (Table 4). Thus, the combination that comes from removing the article from Porter et al. (11) presented better dispersion, and there was no statistically significant superiority between the groups (log OR 0.24, CI 95% −0.80 to 1.28, and *p* = 0.65) with very low heterogeneity (*p* = 0.18 and *I*^2^ = 38.64%), favoring surgical treatment (Figure 3D). By analyzing the funnel plot of the same parameter and removing the previously mentioned manuscript, our study results demonstrated better dispersion, with very low publication bias, as compared when performed including all the manuscripts (Figures 3C,F).

**Table 4:** Statistical characteristics from leave-one-out method.

		**Number of removed trials**	**Name of removed trials**	**Sample size**	**Overall effect**			**Heterogeneity**	
					**Effect estimate**	**CI 95%**	***P*- value**	***I*^2^ (%)**	***P*- value**
1	Rebleeding	–	–	396	0.18	−0.40 to 0.77	0.54	74.47	0.00
	Reintervention				−0.64	−1.64 to 0.37	0.22	62.60	0.02
2	**Rebleeding**	**1**	**Porter et al**. **(****11****)**	**307**	**0.24**	**−0.80 to 1.28**	**0.65**	**38.64**	**0.18**
	Reintervention				0.10	−1.61 to 1.82	0.91	21.59	0.27
3	Rebleeding	1	Mathiesen et al. (9)	369	−0.51	−2.33 to 1.30	0.58	79.28	0.00
	Reintervention				−0.51	−2.78 to 1.76	0.66	66.21	0.01
4	Rebleeding	1	Lunsford et al. (23)	330	−0.99	−2.73 to 0.76	0.27	66.92	0.01
	Reintervention				−0.51	−2.89 to 1.87	0.67	65.21	0.01
5	Rebleeding	1	Kida et al. (22)	248	−0.66	−2.42 to 1.10	0.46	80.75	0.00
	Reintervention				−1.24	−3.14 to 0.65	0.20	51.36	0.08
6	Rebleeding	1	Frischer et al. (20)	349	0.57	−1.24 to 2.38	0.54	61.23	0.02
	**Reintervention**				**−1.29**	**−3.22 to 0.64**	**0.19**	**48.02**	**0.10**
7	Rebleeding	1	Haciyakupoglu et al. (21)	377	−0.41	−2.09 to 1.26	0.63	79.15	0.00
	Reintervention				−0.48	−2.81 to 1.85	0.69	65.60	0.01
8	Rebleeding	2	-Porter et al. (11) -Mathiesen et al. (9)	286	0.70	−0.00 to 1.41	0.05	0.00	0.30
	Reintervention				0.39	−1.61 to 2.39	0.70	32.86	0.21
9	Rebleeding	2	-Porter et al. (11) - Lunsford et al. (23)	241	−0.21	−1.74 to 1.33	0.79	42.50	0.17
	Reintervention				0.51	−1.59 to 2.60	0.64	28.72	0.24
10	Rebleeding	2	-Porter et al. (11) - Kida et al. (22)	159	0.23	−1.00 to 1.46	0.72	52.34	0.11
	Reintervention				−0.38	−2.11 to 1.36	0.67	10.99	0.38
11	Rebleeding	2	-Porter et al. (11) - Frischer et al. (20)	260	−0.19	−1.52 to 1.15	0.78	44.01	0.16
	Reintervention				−0.51	−2.26 to 1.23	0.57	0.00	0.35
12	Rebleeding	2	-Porter et al. (11) Haciyakupoglu et al. (21)	288	0.57	−0.25 to 1.38	0.18	14.64	0.27
	Reintervention				0.50	−1.52 to 2.52	0.63	27.74	0.24
13	Rebleeding	2	-Mathiesen et al. (9) - Lunsford et al. (23)	303	−0.98	−3.22 to 1.26	0.39	73.08	0.00
	Reintervention				−0.27	−3.18 to 2.64	0.86	73.86	0.01
14	Rebleeding	2	-Mathiesen et al. (9) - Kida et al. (22)	221	−0.61	−2.85 to 1.63	0.59	85.66	0.00
	Reintervention				−1.17	−3.43 to 1.08	0.31	62.33	0.04
15	Rebleeding	2	-Mathiesen et al. (9) - Frischer et al. (20)	322	−1.05	−3.11 to 1.01	0.32	75.83	0.00
	Reintervention				−1.16	−3.54 to 1.22	0.34	61.33	0.05
16	Rebleeding	2	-Mathiesen et al. (9) Haciyakupoglu et al. (21)	350	−0.28	−2.38 to 1.82	0.79	84.12	0.00
	Reintervention				−0.23	−3.08 to 2.62	0.87	74.37	0.01
17	Rebleeding	2	- Lunsford et al. (23) - Kida et al. (22)	182	−1.15	−3.26 to 0.95	0.28	74.71	0.00
	Reintervention				−1.21	−3.60 to 1.18	0.32	61.22	0.04
18	Rebleeding	2	- Lunsford et al. (23) - Frischer et al. (20)	283	−1.80	−3.25 to −0.35	0.01	30.10	0.27
	Reintervention				−1.19	−3.73 to 1.35	0.36	60.02	0.05
19	Rebleeding	2	- Lunsford et al. (23) Haciyakupoglu et al. (21)	311	−0.82	−2.88 to 1.24	0.43	74.40	0.00
	Reintervention				−0.23	−3.23 to 2.76	0.88	72.69	0.01
20	Rebleeding	2	- Kida et al. (22) - Frischer et al. (20)	201	−1.20	−3.14 to 0.74	0.23	77.79	0.00
	Reintervention				−2.28	−3.71 to −0.85	0.00	4.60	0.54
21	Rebleeding	2	- Kida et al. (22) Haciyakupoglu et al. (21)	229	−0.47	−2.51 to 1.57	0.65	85.70	0.00
	Reintervention				−1.16	−3.50 to 1.19	0.33	61.96	0.04
22	Rebleeding	2	-Frischer et al. (20) Haciyakupoglu et al. (21)	330	−0.89	−2.77 to 1.00	0.36	77.19	0.00
	Reintervention				−1.16	−3.50 to 1.19	0.33	61.96	0.04

*I^2^ shows the percentage of total variation across studies that is due to heterogeneity rather than chance; CI, confidence interval. The meaning of the bold values provided that when we examined all possible combinations by removing one or two studies at the time, these are the combinations without heterogeneity, with the higher sample size and the less article removed. Thus these combinations displayed better dispersion, with very low publication bias*.

### Late Rebleeding

**Figure 4:**
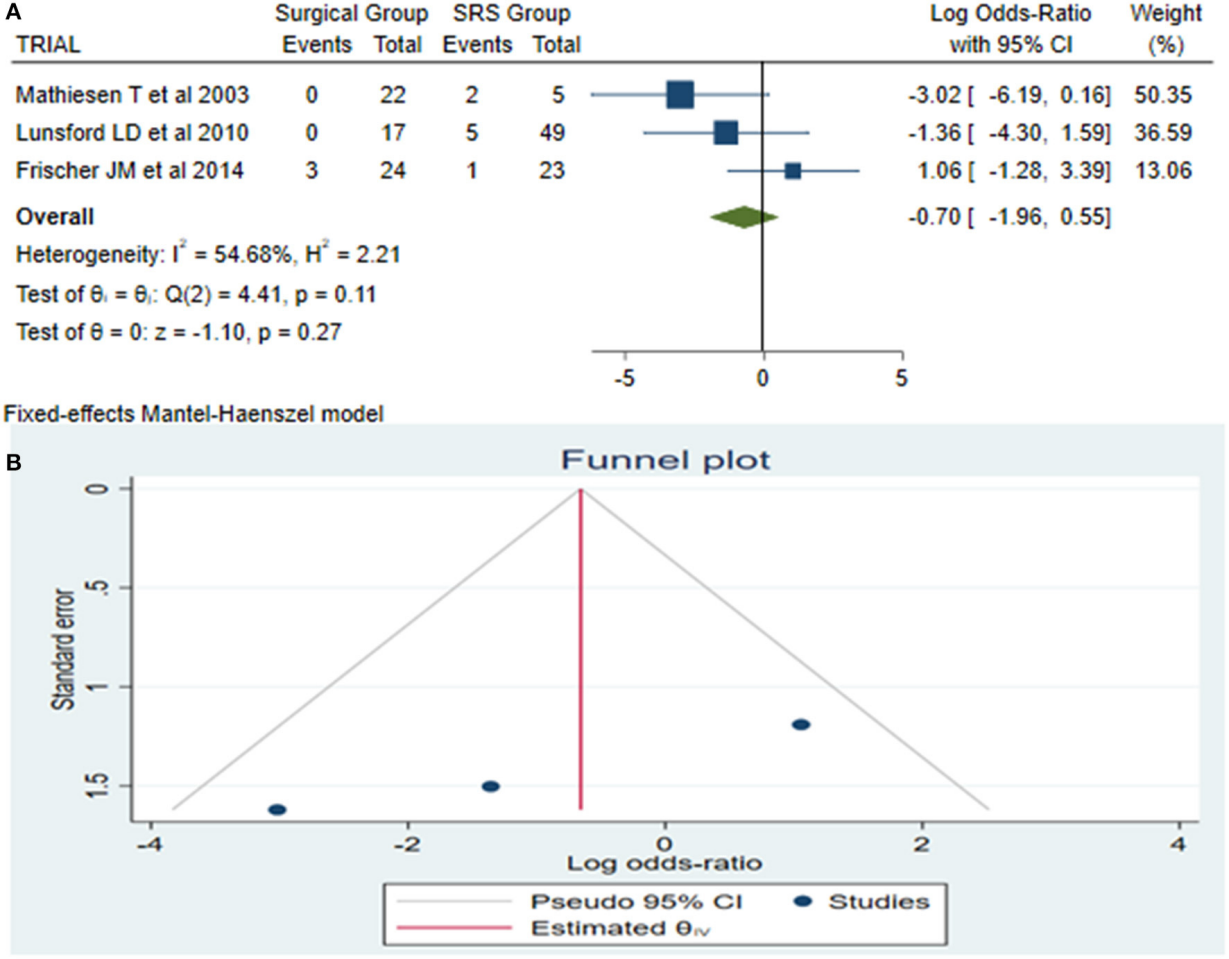
Late rebleeding (LaReBl): **(A)** OR forest plot LaReBl: Results demonstrate no statistically significant difference between the two groups [decimal logarithm of odd ratio—(log OR −0.70, CI 95% −1.96–0.55, and *p* = 0.27), with heterogeneity (*p* = 0.11 and *I*^2^ = 54.68%)], and **(B)** funnel plots of the LaReBl demonstrated no publication bias. SRS, Radiosurgical group; *I*^2^ shows the percentage of total variation across studies that is due to heterogeneity rather than chance; CI, confidence interval.

Late rebleeding was reported by 3 articles with a total of 11 patients (surgical group = 3 patients with subtotal resection and SRS group = 8 patients). The results of the analysis showed no statistically significant difference between the two groups (log OR−0.70, CI 95% −1.96–0.55, and *p* = 0.27**)**, with heterogeneity (*p* = 0.11 and *I*^2^ = 54.68%; Figures 4A,B).

### Reintervention

**Figure 5:**
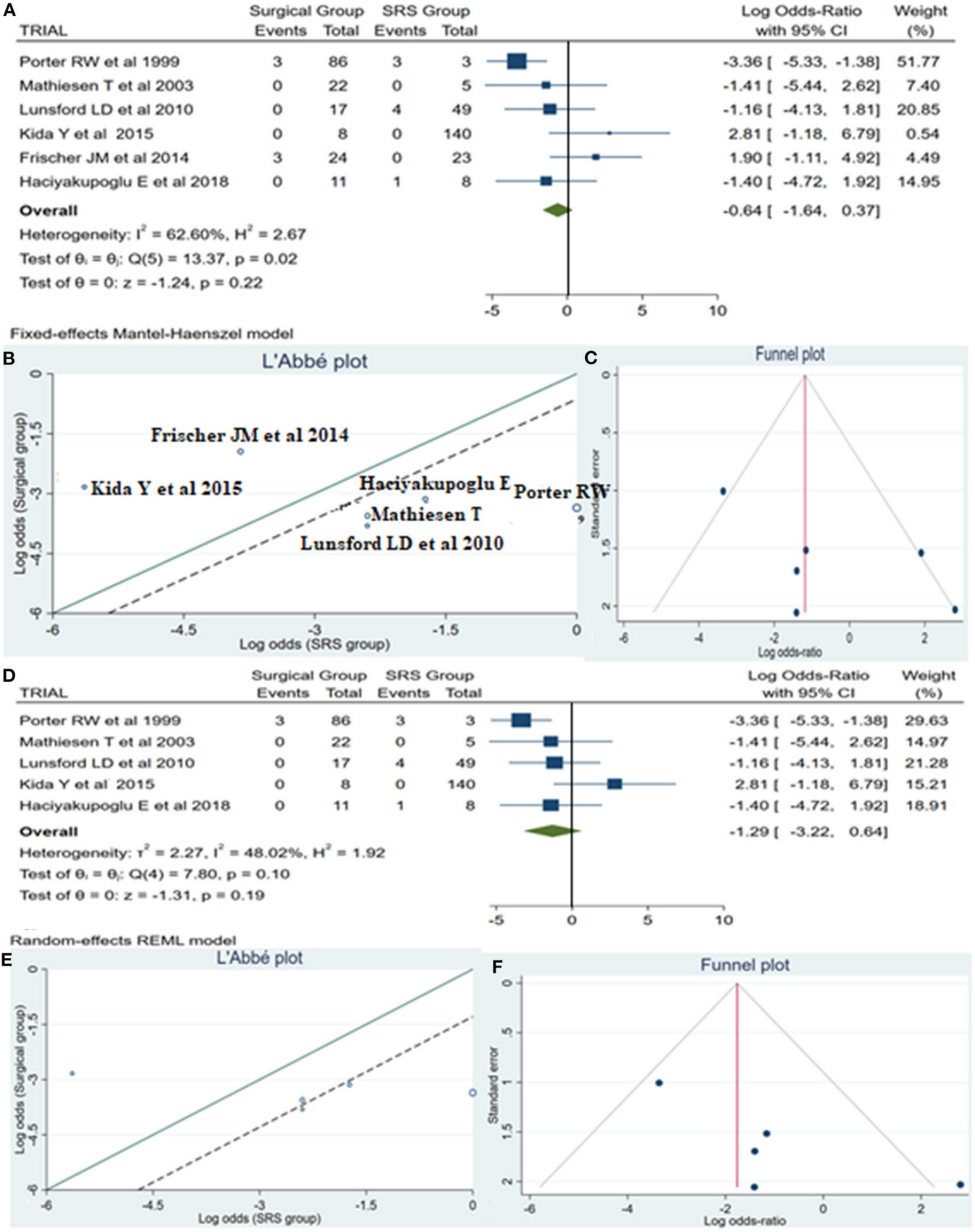
Reintervention (ReInt): **(A)** OR forest plot ReInt: Results demonstrate no statistically significant difference between the two groups (log OR −0.64, CI 95%[(−1.64 to 0.37)], *p* = 0.22), but providing heterogeneity (*I*^2^ = 62.60% and *p* = 0.02); **(B)** L'Abbé plot and analysis of heterogeneity (*p* < 0.1 and *I*^2^ = 62.60%); **(C)** Funnel plots for publication bias on the available evidence relevant to BSCMs management; **(D)** OR forest plot ReInt after sequential removal of “Frischer JM” (20) article demonstrated additionally no statistically significant superiority over two methods (log OR −1.29, CI 95%[(−3.22)−0.64], *p* = 0.19); **(E,F)** L'Abbé and funnel plots demonstrated low heterogeneity (*p* = 0.10 and *I*^2^ = 48.02%) and no publication bias of the ReInt in groups of patients with BSCMs, respectively. SRS, Radiosurgical group; *I*^2^ shows the percentage of total variation across studies that is due to heterogeneity rather than chance; CI, confidence interval.

A total of 14 patients required reintervention, among them 6 patients belonged to the surgical group and the remaining 8 patients to the SRS group. The statistical analysis showed no potentially significant difference between the two groups, with the superiority of the SRS method over surgical treatment (log OR −0.64, CI 95% −1.64 to −0.37, *p* = 0.22, *p* = 0.22), but providing heterogeneity (*I*^2^ = 62.60% and *p* = 0.02) (Figure 5A). As sequential removal of studies one by one and looking at the L'Abbé plot (Figures 5B,E), we examined all possible combinations by removing one or two studies at the time and choose the combination without heterogeneity with the higher sample size and the less article removed (Table 4). Thus, the combination that comes from excluding the study from Frischer et al. (20) showed no statistically significant difference over the two methods, associated with low heterogeneity (*p* = 0.10 and *I*^2^ = 48.02%; Figure 5D). The funnel plot of the same parameter, excluding the previously mentioned study, displayed better dispersion, with very low publication bias, as compared when performed including all the manuscripts (Figures 5C,F).

### Mortality

**Figure 6:**
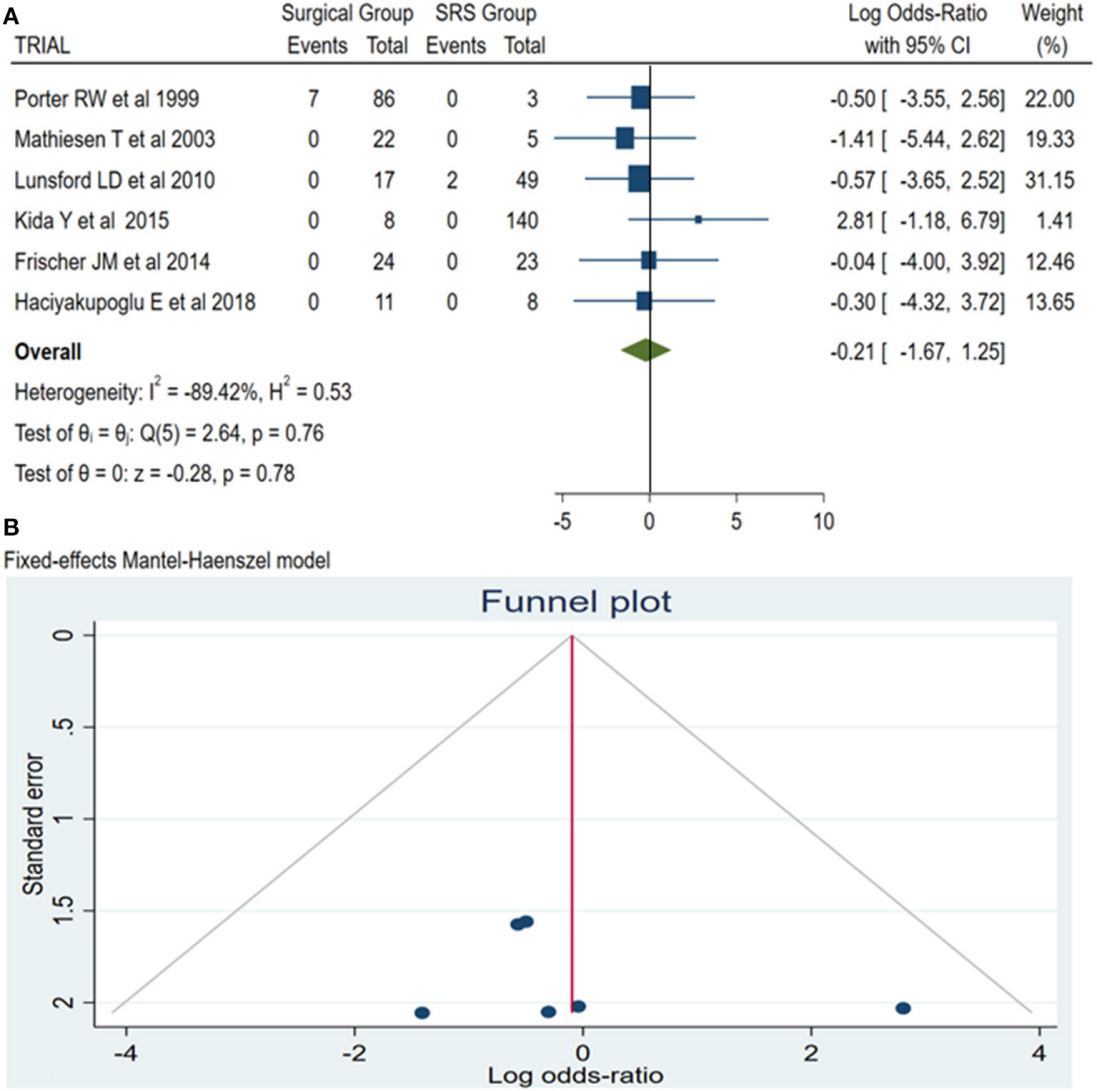
Mortality: **(A)** OR forest plot mortality: Results demonstrate no statistically significant difference between the two groups (log OR −0.21, CI 95% 1.25–1.67, and *p* = 0.78) with no heterogeneity (*p* = 0.76 and *I*^2^ = −89.42%); **(B)** Funnel plot of the same parameter demonstrated no publication bias. SRS, Radiosurgical group; *I*^2^ shows the percentage of total variation across studies that is due to heterogeneity rather than chance; CI, confidence interval.

Mortality reports were available from all retrieved articles. Of this pool of articles, we identified a total of nine dead patients, seven in the surgical group and two within the SRS group. The results of this subanalysis showed no significant difference between the two groups (log OR −0.21, CI 95% −1.67 to 1.25, and *p* = 0.78) with no heterogeneity (*p* = 0.76 and *I*^2^ = −89.42%; Figure 6).

The optimal treatment strategy for BSCMs remains controversial. Microsurgical resection described as the first treatment modality for BSCMs has experienced marked improvement during the last decades.

## Discussion

Brainstem cavernous malformations are challenging and interesting vascular lesions, with a higher risk of bleeding as compared to their supratentorial counterpart, when presented with ICH or FND (7). Furthermore, repeated hemorrhages of these lesions can be devastating and may cause severe and irreversible neurological deficits. Therefore, it requires adequate management to decrease rebleeding rates and unexpected events.

On the contrary, during the last decades, SRS emerged as an alternative treatment modality to reduce the risk of rebleeding of those surgically inaccessible lesions and among patients with severe comorbidities (16, 18, 23, 26). However, its long-term effects have been debatable.

Moreover, pooling data from individual series according to a systematic review and meta-analysis enable and improve to understand the potentials of each treatment modality while managing BSCMs.

### Morbidity

Surgically treated BSCMs have a rate of temporary and/or permanent morbidity of up to 35–69% (9, 11). Among these patients, around 5–12% suffer from PND, which will outweigh the morbidity and mortality rates associated with untreated symptomatic lesions over time, provided that surgery reaches its goal of radical removal (9, 11).

On the other hand, until now, SRS has not been directly associated with PND, and the majority of complications or comorbidities related to this procedure are radiation-induced adverse events, which generally are transitory and respond to short-duration treatment with corticosteroids (14, 15, 17). The incidence of radiation-induced complications after SRS in BSCMs is higher as compared to SRS of arteriovenous malformations, maybe due to the reason that parenchyma surrounds the CM, which contains hemosiderin that could act as a radiation sensitizer. Initially, the rate of complications after SRS ranged around 59%; however, during the last decade, it decreased from 9.1 to 0% on low radiation dose applications, which could be related to better planning, improvements in technology, and experience (27, 28).

Our study showed no superiority among PND between the microsurgery- and the SRS-treated patients.

### Rebleeding Rate

The main goal of treatment is to eliminate the risk of bleeding, which can cause severe and irreversible neurological deficits. Microsurgical treatment immediately eliminates this risk; however, it requires complete and safe radical resection of the lesion. Since a small percent (~2.5%) of poor outcomes is due to rebleeding from residual cavernomas (9, 29). Moreover, SRS for BSCMs apparently reduces the risk of hemorrhage from ~30 to 10.8% per lesion per year within the first 2 years after treatment, and to an annual rate of 1.1% thereafter, which seems to be beneficial due to the high risk of bleeding and poor outcome (34% moderately-to-severely disability or dead) among conservative managed patients (8, 26). This is not in concordance with our results, since SRS demonstrated no superiority over surgical treatment when comparing pooled data. One explanation may be that in many studies used a restrictive definition of “overt bleeding” as hemorrhage outside the confines of the lesion and because of the brainstem's densely packed neural functions, even very small hemorrhages are likely to elicit symptoms and thus were found an astonishing high figure of hemorrhages (≈88%) (8). A less probable explanation would be that BSCMs in the brainstem are associated with a higher risk of hemorrhage, where the rebleeding rate among all possible treatment modalities is approximately the same and should represent a separate entity.

### Mortality and Reintervention

In our study, no statistically significant difference or superiority existed between surgical treatment or SRS of symptomatic BSCMs regarding mortality rate, which is in line with previous studies that reported surgical treatment associated with overall 30-day mortality between 3.5 and 0.8%, similar to the values described of 3% following stereotactic Bragg peak proton beam therapy (9, 11, 29, 30).

Regarding the reintervention rate, initially, we identified some significant statistical predominance of SRS over surgical treatment. However, due to heterogeneity, we excluded two large case series [From Porter et al. (11) and Lunsford et al. (23)] which show no significant difference between both treatment procedures.

Patients with remnant (after subtotal resection) BSCMs after surgical treatment and patients with failed SRS treatment that showed no radiographic modifications in follow-ups and that suffered recurrent rehemorrhages are usually subjected to reintervention procedures.

Based upon the limited available evidence and the findings of this meta-analysis, surgery may confer a benefit to patients with BSCMs and should be considered as the first-line management especially of BSCM with progressive neurologic deficits, extralesional or intralesion hemorrhage with mass effect, exophytic type, younger age, and small lesions (31). However, we agreed that surgical management for asymptomatic deep BSCM and patients with severe comorbidities need a meticulous evaluation. Conservative management or current SRS strategy of using a lower dose of radiation (<15 Gy) may become the feasible options for this group of patients (32, 33).

### Limitations

Despite the efforts of the authors on the selection of proper and eligible studies, there is still the possibility of selection bias. At the present, the majority of patients treated by SRS are those patients with contraindications for microsurgery or harboring residual CMs. In addition, because the articles reviewed were retrospective and size of the case series was relatively small, we cannot expect high level of evidence from the literature review and meta-analysis on this topic.

## Conclusion

Microsurgical treatment of BSCMs immediately eliminates the risk of rehaemorrhage; however, it requires complete excision of the lesion and it is associated with a similar rate of PND compared with SRS management. Apparently, SRS of BSCMs causes a marked reduction in the risk of rebleeding 2 years after treatment. Both treatment procedures have similar 30-day mortality and reintervention rates.

## Author Contributions

GF and HA-B contributed in acquisition of data, performed analysis and interpretation of data, and provided administrative or technical or material support. GF, HA-B, JK, MT, FG, and JH drafting the article. GF approved the final version of the manuscript on behalf of all authors and contributed in statistical analysis. JH, GF, and HA-B supervised the study. All authors contributed in conception and design, critically revised the article, and agreed to be accountable for the contents of this work.

## Conflict of Interest

The authors declare that the research was conducted in the absence of any commercial or financial relationships that could be construed as a potential conflict of interest.

## Publisher's Note

All claims expressed in this article are solely those of the authors and do not necessarily represent those of their affiliated organizations, or those of the publisher, the editors and the reviewers. Any product that may be evaluated in this article, or claim that may be made by its manufacturer, is not guaranteed or endorsed by the publisher.
